# Gaps in Knowledge About SARS-CoV-2 & COVID-19 Among University Students Are Associated With Negative Attitudes Toward People With COVID-19: A Cross-Sectional Study in Cyprus

**DOI:** 10.3389/fpubh.2021.758030

**Published:** 2021-11-19

**Authors:** Nicos Middleton, Constantinos Tsioutis, Ourania Kolokotroni, Alexandros Heraclides, Panagiotis Theodosis-Nobelos, Ioannis Mamais, Maria Pantelidou, Dimitrios Tsaltas, Eirini Christaki, Georgios Nikolopoulos, Nikolas Dietis

**Affiliations:** ^1^Department of Nursing, School of Health Sciences, Cyprus University of Technology, Limassol, Cyprus; ^2^School of Medicine, European University Cyprus, Nicosia, Cyprus; ^3^Department of Primary Care and Population Health, Medical School, University of Nicosia, Nicosia, Cyprus; ^4^Department of Health Sciences, School of Sciences, European University Cyprus, Nicosia, Cyprus; ^5^Department of Pharmacy, School of Health Sciences, Frederick University, Nicosia, Cyprus; ^6^Department of Agricultural Sciences, Biotechnology and Food Science, Cyprus University of Technology, Limassol, Cyprus; ^7^Medical School, University of Cyprus, Nicosia, Cyprus

**Keywords:** COVID-19, SARS-CoV-2, student, coronavirus, universities, knowledge, attitudes, perceptions

## Abstract

University students represent a highly active group in terms of their social activity in the community and in the propagation of information on social media. We aimed to map the knowledge, attitudes, and perceptions of University students in Cyprus about severe acute respiratory syndrome coronavirus-2 (SARS-CoV-2) and Coronavirus disease 2019 (COVID-19) to guide targeted future measures and information campaigns. We used a cross-sectional online survey targeting all students in conventional, not distance-learning, programs in five major universities in the Republic of Cyprus. Students were invited to participate through the respective Studies and Student Welfare Office of each institution. The survey was made available in English and Greek on REDCap. Participation was voluntary and anonymous. The questionnaire was developed based on a consensus to cover the main factual information directed by official channels toward the general public in Cyprus at the time of the survey. In addition to sociodemographic information (*N* = 8), the self-administered questionnaire consisted of 19 questions, assessing the knowledge regarding the characteristics of SARS-CoV-2 and COVID-19, infection prevention and control measures (*N* = 10), perceptions related to COVID-19, for instance, whether strict travel measures are necessary (*N* = 4), and attitudes toward a hypothetical person infected (*N* = 2). Furthermore, participants were asked to provide their own assessment of their knowledge about COVID-19 and specifically with regard to the main symptoms and ways of transmission (*N* = 3). The number of students who completed the survey was 3,641 (41% studying Health/Life Sciences). Amongst them, 68.8% responded correctly to at least 60% of knowledge-related questions. Misconceptions were identified in 30%. Only 29.1% expressed a positive attitude toward a hypothetical person with COVID-19 without projecting judgment (9.2%) or blame (38%). Odds of expressing a positive attitude increased by 18% (95% CI 13–24%; *p* < 0.001) per unit increase in knowledge. Postgraduate level education was predictive of better knowledge (odds ratio (OR) 1.81; 95% CI 1.34–2.46; *p* < 0.001 among doctoral students] and positive attitude [OR 1.35; 95% CI 1.01–1.80; *p* = 0.04). In this study, we show that specific knowledge gaps and misconceptions exist among University students about SARS-CoV-2 and COVID-19 and their prevalence is associated with negative attitudes toward people with COVID-19. Our findings highlight the integrated nature of knowledge and attitude and suggest that improvements to the former could contribute to improvements in the latter.

## Introduction

Coronavirus disease 2019 (COVID-19) is a novel respiratory infection caused by the severe acute respiratory syndrome Coronavirus-2 (SARS-CoV-2). It quickly spread across the world, making the WHO declare the situation a public health emergency of international concern on January 30, 2020, and a pandemic on March 11, 2020 (1–4).

An important aspect in the attempt to control the spread is to ensure the understanding of the public with regard to the transmission of the virus, the symptoms of the disease, and protective measures. The main symptoms include fever, cough, fatigue, myalgia, nausea, and shortness of breath, which in severe cases may lead to acute respiratory distress syndrome or cardiac injury (5). Severe forms of infection concern mainly people with comorbidities, immunosuppression, and the elderly (6). Transmission of the virus is done mainly through respiratory droplets. Transmission precautions are of critical importance, including physical distancing, wearing of masks, hand hygiene, and respiratory etiquette, particularly considering the longer incubation time of the virus.

The Republic of Cyprus, with a population of 888,000, reported its first two cases of COVID-19 on March 9, 2020. On March 21st, the first death was recorded (7). The government of Cyprus adopted restrictive measures to prevent transmission, including movement prohibitions, compulsory distancing, and self-isolation between March 24, 2020, and May 3, 2020 (8). In addition to the WHO and the European Center for Disease Prevention and Control (ECDC), the main local sources of information for the public were the press releases by the Ministry of Health through the Press and Information Office (8).

More than 38,000 students were enrolled in the tertiary education institutions in the Republic of Cyprus, accounting for nearly 5% of the total population (9). Due to their high social networking and general mobility, students play an important role in the spread of communicable diseases. Students are also known to be highly active in social media platforms and the internet, rendering them an important group when it comes to the propagation of information during crises. Moreover, they are members of families and are therefore potential spreaders of viral infections to vulnerable population groups.

The aim of the current study was to investigate the University students knowledge and perception of SARS-CoV-2 and COVID-19. Identifying misinformation and misperceptions in this group is important in the context of customizing the content of communication material and other information activities. Several studies in the literature have assessed knowledge, attitudes, perceptions, and/or practices related to COVID-19, some directed toward the general population and others toward specific population groups, including University students. Often, studies among University students originate from a single academic institution directed toward its own student body. While findings in such cross-sectional studies are time-bound and can only be understood within the local context, the assessment of knowledge and attitudes of University students is important, especially when originating directly from the institutions involved, which have a responsibility to understand the current level of affairs among their community and thus tailor their educational and community-building efforts accordingly and expand the various channels of communication with the students. Through the COVID-19 interacts partnership across all five major universities in Cyprus, this study was addressed to the nationwide population of University students.

## Methods

This was a cross-sectional, multicenter, web-based survey across the five major universities in the Republic of Cyprus [two state-funded: University of Cyprus (UCY), Cyprus University of Technology (CUT) and three private: European University Cyprus (EUC), University of Nicosia (UNIC), and Frederick University (FrU)]. The self-administered survey was released in English and Greek from March 17 to 31, 2020. Due to the need to gather quick “intelligence” about potential misconceptions and/or problematic attitudes among the student population, as the response to the pandemic in Cyprus was unfolding and the country was entering its first lockdown, it was decided in advance that the survey will remain open for a period of 2 weeks (17–31 March), unless there was a need to extend the survey further due to suboptimal participation. It was constructed, delivered, and managed using REDCap (10), installed in secure servers of UCY, and technically supported by UCY Information and Technology Department to ensure data integrity.

Following institutional approval by participating universities and the Cyprus National Bioethics Committee (approval no. 2020.01.51), a password-protected weblink was forwarded to the institutional email of the potential participants. Inclusion criteria were as follows: the student should (a) be registered at any of the five participating academic institutions, with an active email institutional address; (b) follow a conventional, not distance-learning, program of study, irrespective of the academic field, department, or faculty; (c) give consent, and finally to participate by checking the informed consent opt-in box provided at the beginning of the survey; and (d) respond to the full list of questions in the online questionnaire.

Participation was voluntary and anonymous, after informed consent, provided in the form of a declaration checkbox prior to opting in. Participants could cancel their interaction at any time by exiting the web browser prior to submission. Students were invited to participate through the respective Studies and Student Welfare Office of each institution. A presurvey section included confirmation of the status of the participating student by stating their institutional email. Students enrolled in conventional undergraduate and postgraduate programs were eligible to participate, irrespective of the school/department and the level of study (undergraduate, postgraduate, and doctorate), ensuring a diversity of background and covering a long list of academic subjects offered across these universities. Distance learning students were not included, since the focus was on perceptions among those residing in Cyprus at the early stages of the outbreak. The total number of eligible students invited to participate was 19,176.

The survey questionnaire covered knowledge and practices, self-assessment of their knowledge, perceptions, and attitudes. Due to the topic under investigation (i.e., an epidemic of a new disease with its own special characteristics), the survey did not include previously validated questionnaires or a set of questions, but questions developed by consensus of all study investigators to capture the local experience based on the main factual information directed by official channels toward the general public in Cyprus at the time of the survey. The penultimate draft was pilot-tested by study investigators to provide feedback on the technical aspects, user-friendliness of the interphase, operationality, and handling. Necessary modifications were applied before finalizing the survey.

The questionnaire consisted of 27 questions across four sections: 8 on sociodemographic characteristics and study-related information; 10 on knowledge about the characteristics of SARS-CoV-2 and COVID-19, infection prevention, and control measures; 4 on general perceptions related to COVID-19; 2 on attitudes toward a hypothetical person infected; 1 on self-assessment of the general level of knowledge about COVID-19 and 2 on specific knowledge with regard to the main symptoms and ways of transmission (Survey Questionnaire blueprint, Supplementary Figure 1).

The number of items included in the measurement tools to capture the knowledge of the University students may range in numbers across similar studies; nevertheless, all tools tend to be short, for instance, from 14 items, as in Khasawneh et al. (11) to 23 items as in Lincango-Naranjo (12), covering common areas, such as mode of transmission, virulence, main symptoms, vulnerable groups, and preventative measures. Across studies, items are selected based on the national and international guidelines (WHO, CDC, ECDC), while keeping the questionnaire short. In the case of our study, multiple choice responses with one or several answers (for example, “Which of the following are the vulnerable groups?”) were preferred rather than True vs. False statements, commonly included in other knowledge surveys (for example, “People with chronic diseases are at a higher risk.”) (11, 12).

A “Positive attitude” toward a person infected with COVID-19 was operationalized using two definitions: (a) positive response to statements “doesn't change the way I think about them” and “I will try to support them as much as I can,” irrespective of other statements in the response, and (b) subsample of participants who responded positively to these statements and also negatively to statements, such as “the person has bad self-hygiene” or “they were negligent to self-protect efficiently.” In the questionnaire, students also had to denote whether their “degree is related to Health of Life Sciences e.g., Medicine, Nursing, Pharmacy, Biology, Microbiology, Public Health, etc.” Based on the response, the participants were classified into two groups (Health/Life Sciences vs. the rest) for the purpose of the analysis. Students in Health or Life Science programs were further asked whether epidemic management and Public Health protection were part of their curriculum. Upon submission of the questionnaire, students were presented with an information page with the correct answers to the knowledge questions, based on the WHO guidelines at the time.

Characteristics of the participants are presented in the overall sample and by field of study (Health/Life sciences vs. the rest). Differences in self-assessed knowledge were assessed in parametric (one-way ANOVA and *t*-test for independent samples) and non-parametric (Kruskal–Wallis) tests according to demographic characteristics and field of study. Differences in knowledge, perception, and attitudes between Health or Life Science students vs. the rest were explored in chi-squared tests. In addition to responses to each individual question, an overall knowledge score with a theoretical range of 0–10 was calculated as the sum of the correct answers (single correct answer or combination of answers, as applicable) assuming equal weight across all 10 questions. Predictors of better knowledge operationalized as continuous and as categorical variables (score of ≥8) were explored in linear and binary logistic stepwise regression models, respectively. All demographic, perception, and attitude variables were considered in the multivariable regression models. Logistic regression models were also used to estimate the odds of a positive attitude toward an infected person by knowledge, perceptions, and sociodemographic variables.

## Results

A total of 3,641 complete responses were received (65.5% women, 80.9% bachelors, and 85.6% native Greek speakers); response rate of 19% (Table 1). In total, two-fifths (41.1%) were enrolled in Health or Life Sciences programs. Among those, 61.9% reported that epidemic response was part of their curriculum program. The large sample size ensures high precision in the estimates. For example, precision analysis indicates that percentage responses around 50% are estimated with a margin of error smaller than 2% while for lower (e.g., 5%) and higher (e.g., 90%) percentages, the 95% CI have an even smaller margin of error (<1%). Furthermore, *post-hoc* power analysis indicates that the study had at least 95% of power to detect differences between comparison groups as statistically significant at the 5% of as little as 0.2 in the case of continuous variables and smaller than 5% of point differences in the case of categorical variables.

**Table 1:** Sociodemographic characteristics of the participants and self-evaluation of the level of knowledge about new coronavirus/COVID-19 across five universities and according to study program (*N* = 3,641, listwise complete responses).

	**All (*N =* 3,641)**	**Study program**			**Self-evaluation of knowledge (0–100 scale)**	
		**Health and life sciences**	**Other^a^**	***P*-value^b^**	**Median (IQR)**	***P*-value^c^**
		**(*N =* 1,496, 41.1%)**	**(*N =* 2,145, 58.9%)**			
**Gender**						
Female	2,384 (65.5%)	69.5%	62.7%	<0.001	75 (65–85)	<0.001
Male	1,247 (34.3%)	30.3%	37.0%		80 (70–90)	
Other	10 (0.3%)	0.2%	0.3%		66 (50–70)	
**Study level**						
Bachelor	2,945 (80.9%)	81.8%	80.3%	<0.001	77 (65–85)	0.27
Master's	399 (11.0%)	8.6%	12.6%		78 (66–86)	
Doctoral	224 (6.2%)	6.0%	6.3%		77 (68–90)	
Other	73 (2.0%)	3.7%	0.8%		80 (70–87)	
**Native language**						
Greek	3,115 (85.6%)	80.7%	89.0%	<0.001	76 (65–85)	0.004
Other	526 (14.5%)	19.3%	11.1%		80 (70–90)	
**Epidemic management/ Public Health response (*****N =*** **1,496)**						
Yes		926 (61.9%)			80 (70–90)	<0.001
No		570 (38.1%)			75 (62–85)	
**Health professionals' first-degree relatives**						
Yes	1,163 (31.9%)	43.2%	24.1%	<0.001	80 (69–88)	0.003
No	2,365 (65.0%)	54.6%	72.2%		76 (65–85)	
Not know	113 (3.1%)	2.2%	3.7%		75 (61–86)	
**The University will find ways to minimize the impact of the pandemic on your studies**						
Yes	2,830 (77.7%)	80.8%	75.6%	0.001	78 (68–86)	<0.001
No	133 (3.7%)	3.0%	4.1%		79 (65–87)	
Not sure	678 (18.6%)	16.2%	20.3%		75 (61–85)	
**Self-evaluation of knowledge about COVID-19 on a 0–100 scale**						
Mean (SD)	74.7 (16.8)	75.5 (16.5)	74.1 (16.9)	0.01		
Median (IQR)	77 (66–85)	79 (70–87)	76 (65–85)	0.004		

*COVID-19, coronavirus disease 2019; IQR, interquartile range; SD, standard deviation*.

a *Academic study program is not related to Health or Life Sciences, which includes Medicine, Nursing, Pharmacy, Biology, Microbiology, Public Health etc.,*

b *p-value of χ^2^-test*,

c *p-value of Kruskal–Wallis test*.

### Knowledge About SARS-CoV-2 and COVID-19

Responders assessed their own level of knowledge as relatively high, with a mean of 74.7 (SD 16.8) and median 77 [interquartile range (IQR) 66–85] on a 0–100 scale. Differences in self-assessed knowledge by study program and sociodemographic characteristics are presented in Table 1. Even though statistically significant in many cases due to the large sample, differences were too small to be meaningful and only in the range of 2–3 points on average.

In general, 68.8% of the participants responded correctly (based on the scientific knowledge at that time) to at least six questions, and 20.6% responded correctly to eight or more (Table 2). The highest percentages of correct answers were recorded in questions related to washing hands to reduce the risk of infection (true, 96.8%), the maximum time between infection and presentation of symptoms (2 weeks, 84.0%), and local telephone line to contact authorities (1420, 82.3%). A relatively high percentage of correct answers was also recorded in relation to the global case fatality rate (between 1 and 10%, 73.5%), the protective use of surgical masks by healthy individuals (not true, 71.7%), and availability of preventive medicines or vaccines to reduce risk of infection (not true, 71.2%).

**Table 2:** Knowledge about symptoms, virus transmission, personal, and public health response by tertiles of self-assessment of knowledge on a 0–100 scale (*N* = 3,641).

	**All**	**T1 (≤70)**	**T2 (71–82)**	**T3 (83)**	***P*-value**
**A. Three main characteristic symptoms of COVID-19**					
1. Headache	277 (7.6%)	8.9%	6.6%	7.1%	0.08
2. Fever	3,514 (96.5%)	96.1%	97.1%	96.5%	0.37
3. Shortness of breath	2,657 (73.0%)	72.0%	71.9%	75.0%	0.15
4. Chest pain	142 (3.9%)	4.3%	4.2%	3.2%	0.28
5. Cough	3,338 (91.7%)	90.9%	91.5%	92.7%	0.24
6. Diarrhea	38 (1.0%)	1.3%	0.8%	1.0%	0.54
7. Weakness/Fatigue	563 (15.5%)	16.0%	15.1%	15.2%	0.95
8. Runny nose	581 (16.0%)	15.5%	14.8%	16.0%	0.70
**Answered 2, 5, and 7 only^a^**	**313 (8.6%)**	**8.6%**	**8.1%**	**9.0%**	**0.72**
**Answered 2, 3, and 5 only**	**2,290 (62.9%)**	**61.4%**	**62.1%**	**65.3%**	**0.10**
**B. Modes of transmission of the novel coronavirus**					
1. Sexual contact	756 (20.8%)	18.2%	21.1%	23.3%	0.006
2. Droplets sneezing/coughing	3,252 (89.3%)	82.1%	92.4%	94.6%	<0.001
3. Through breathing air	1,067 (29.3%)	28.6%	28.5%	30.9%	0.33
4. Consuming contaminated food	460 (12.6%)	10.1%	14.2%	14.0%	0.002
5. Contact with contaminated surface and touching mouth, eyes, nose	3,033 (83.3%)	76.9%	87.1%	87.0%	<0.001
**Answered 2 and 5 only^b^**	**2,280 (37.4%)**	**34.1%**	**40.3%**	**38.4%**	**0.004**
**C. Maximum time between time of infection and first presentation of symptoms**					
Within 1 month	85 (2.3%)	2.1%	2.1%	2.8%	0.61
**Within 2 weeks**	**3,058 (84.0%)**	**84.5%**	**83.9%**	**83.5%**	
Within 1 week	358 (9.8%)	9.6%	10.7%	9.4%	
Within 2 days	140 (3.9%)	3.9%	3.4%	4.3%	
**D. Average global fatality of COVID-19**					
**Between 1–10%**	**2,676 (73.5%)**	**67.0%**	**76.3%**	**78.2%**	<0.001
Between 10–30%	417 (11.5%)	13.8%	10.7%	9.5%	
Over 30%	128 (3.5%)	3.4%	3.1%	4.0%	
Don't know	420 (11.5%)	15.7%	10.0%	8.3%	
**E. Vulnerable groups, i.e., those with higher risk of getting seriously ill or dying from COVID-19**					
1. Children	562 (15.4%)	16.9%	15.5%	13.8%	0.10
2. People over 65 years old	3,500 (96.1%)	94.4%	97.3%	97.0%	<0.001
3. Pregnant women	1,601 (44.0%)	44.4%	46.3%	41.4%	0.05
4. People with chronic diseases	3,324 (91.3%)	90.1%	92.2%	91.8%	0.13
5. Migrants/Refugees	114 (3.1%)	2.7%	3.6%	3.2%	0.41
6. People with low immune system	3,263 (89.6%)	88.6%	89.9%	90.6%	0.26
**Answered 2, 4, and 6 only**	**1,412 (38.8%)**	**35.7%**	**37.2%**	**43.8%**	**0.001**
**F. Risk of infection reduced by washing hands thoroughly with water and soap**					
**True**	**3,523 (96.8%)**	**95.2%**	**97.5%**	**97.8%**	<0.001
Not true	55 (1.5%)	1.5%	1.5%	1.5%	
Not sure	63 (1.7%)	3.3%	1.0%	0.7%	
**G. Protective measures should be mostly used by the elderly and populations at risk**					
True	1,515 (41.6%)	42.9%	41.1%	40.7%	0.64
**Not true**	**2,037 (56.0%)**	**54.7%**	**56.8%**	**56.6%**	
Not sure	89 (2.4%)	2.5%	2.1%	1.8%	
**H. Preventative medicines or vaccines to reduce potential risk of being infected**					
True	171 (4.7%)	4.7%	4.0%	5.4%	<0.001
**Not true**	**2,593 (71.2%)**	**65.3%**	**71.8%**	**77.4%**	
Not sure	877 (24.1%)	30.1%	24.2%	17.3%	
**I. Use of surgical masks by the healthy is regarded as a necessary protective measure**					
True	605 (16.6%)	17.2%	14.9%	17.6%	<0.001
**Not true**	**2,609 (71.7%)**	**67.9%**	**74.1%**	**73.7%**	
Not sure	427 (11.7%)	15.0%	11.1%	8.7%	
**J. Telephone number to contact authorities**					
**1420**	**2,997 (82.3%)**	**80.3%**	**83.2%**	**83.7%**	0.05
Wrong or Don't remember	644 (17.7%)	19.7%	16.8%	16.3%	
**Sum of correct answers—knowledge score (theoretical range 0–10)**					
Mean (SD)	6.2 (1.6)	5.9 (1.6)	6.3 (1.5)	6.4 (1.5)	<0.001
Median (IQR)	6 (5–7)	6 (5–7)	6 (5–7)	7 (5–7)	<0.001
**Number of correct answers**					
Three or lower	181 (5.0%)	7.3%	3.6%	3.6%	<0.001
Four	370 (10.2%)	12.5%	9.3%	8.4%	
Five	587 (16.1%)	16.9%	18.1%	13.4%	
Six	833 (22.9%)	25.3%	21.2%	21.7%	
Seven	921 (25.3%)	21.0%	26.1%	29.4%	
Eight	525 (14.4%)	12.9%	14.2%	16.3%	
Nine or higher	224 (6.2%)	4.1%	7.4%	7.3%	

*The sections (A–J) correspond to the respective sections as they appeared in the survey*.

*COVID-19, coronavirus disease 2019; IQR, interquartile range; SD, standard deviation*.

a *Percentage of correct answer among those who responded that they knew the three main symptoms (N = 3,271, 89.8%) is 8.2 vs. 16.1% and 12.1% among those who responded that they did not know (N = 31, 0.9%) or were unsure (N = 339, 9.3%), respectively (p = 0.01)*.

b *Percentage of correct answer among those who responded that they knew how the novel coronavirus was transmitted (N = 3,357, 92.2%) is 40.3 vs. 25% and 1.1% among those who responded that they did not know (N = 20, 0.6%) or were unsure (N = 264, 7.3%), respectively (p <0.001)*.

In the case of symptoms, transmission, and vulnerable groups, knowledge appeared more fragmented. For instance, while the vast majority reported that fever (96.5%) and cough (91.7%) are symptoms of COVID-19, only 8.6% included fatigue in the three main characteristic symptoms. Moreover, a high percentage of responders reported correctly that people over 65 years, with chronic conditions, and immunocompromised have a higher risk of serious illness or death from COVID-19. However, only 38.8% included only these three groups in their answer, since the majority also included incorrectly other groups from the list of options. Similarly, while 89.3% reported viral transmission *via* droplets and touching the face after contact with the contaminated surfaces (83.3%), only 37.4% included these two options correctly while the majority included other answers incorrectly, with 20.8%, for example, reporting transmission through sexual contact.

A stepwise association between correct answers and self-assessed knowledge or at least a lower percentage among the tertiles who rated their knowledge lower was observed in seven out of 10 questions. Differences were generally small both in questions answered correctly by most participants (e.g., reduced risk of infection by washing hands, 95.2 vs. 97.5 vs. 97.8%, *p* < 0.001) and in those answered correctly by a smaller percentage (e.g., the transmission of COVID-19, 34.1 vs. 40.3 vs. 38.4%, *p* = 0.004). Even though there was a systematic pattern of generally better performance by Health or Life Science students, differences were too small to be meaningful (average knowledge score 6.4 (SD 1.6) vs. 6.1 (SD 1.6); *p* < 0.001).

### Perceptions, Beliefs, and Attitudes

There were no significant differences between students in Health and Life Sciences vs. other programs in terms of general perceptions and beliefs (Table 3). In terms of disease spread, the optimism that things will be better in Cyprus compared to Europe was marginally higher among Health & Life Science students (40.1% compared to 35.5% in other programs; *p* < 0.005). Nevertheless, while 85% reported that their relationship with a person having COVID-19 would not change, 37.6% described this hypothetic person as “negligent to protect themselves efficiently,” and 33.4% did not state that they would “try to support this person as much as they can.” The percentage who expressed a positive attitude without also projecting judgment was only 28–29%, which was surprisingly similar among Health or Life Science and other students.

**Table 3:** Perceptions, beliefs, and attitudes by study program (*N* = 3,641) and whether curriculum covered epidemic management (*N* = 1,496).

	**Study program**			**Curriculum: Epidemic management/PH response^c^**		
	**H & L**	**Other**	***p*-value**	**Yes**	**No**	***p*-value**
	**(*N =* 1,496)**	**(*N =* 2,145)**		**(*N =* 926)**	**(*N =* 570)**	
**Knowledge score (theoretical range 0–10)**						
Mean (SD)	6.4 (1.6)	6.1 (1.6)	<0.001	6.4 (1.6)	6.3 (1.5)	0.14
**Perceptions and Beliefs**						
*Know a person who takes antibiotics as a means of prevention*						
Yes	2.8%	2.6%	0.65	3.0%	2.5%	0.52
*Strict travel restrictions a necessary measure for reducing rates of new cases*						
They are necessary	92.9%	93.1%	0.87	93.1%	92.6%	0.74
*Spread in Cyprus, relative to its population, in comparison with Europe*						
More	23.1%	27.1%	0.005	22.5%	24.0%	0.009
Same	36.8%	37.4%		34.5%	40.7%	
Less	40.1%	35.5%		43.1%	35.3%	
*Reasons to call the State number 1420*						
Symptoms of infection	76.0%	76.2%	0.90	74.8%	77.9%	0.18
Symptoms for >7 days	55.6%	55.6%	0.99	55.3%	55.0%	0.80
Someone I know has symptoms	35.6%	36.9%	0.44	35.3%	36.1%	0.75
Symptoms and traveled abroad	90.1%	89.5%	0.56	89.0%	91.9%	0.06
Informed about symptoms	13.8%	10.8%	0.006	14.7%	12.5%	0.23
Informed about transmission	11.9%	9.1%	0.007	12.6%	10.7%	0.26
Medical emergency	30.2%	32.3%	0.18	28.9%	32.1%	0.20
**Attitudes**						
*Affect relationship with or opinion for a person with COVID-19*						
Neither relationship/opinion	86.4%	86.1%	0.73	87.7%	84.2%	0.12
Not opinion, but relationship	11.1%	11.0%		10.3%	12.5%	
Relationship and opinion	2.5%	3.0%		2.1%	3.3%	
*A person you know has been infected. Which statements describe your opinion best*						
1. Bad self-hygiene	9.2%	7.8%	0.16	9.0%	9.5%	0.74
2. Negligent to self-protect	38.0%	37.3%	0.71	38.0%	37.9%	0.96
3. Avoid conduct in future	12.2%	12.8%	0.61	11.5%	13.3%	0.28
4. Doesn't change way I think	78.5%	77.2%	0.36	78.4%	78.6%	0.93
5. Try to support them	66.6%	62.8%	0.02	67.7%	64.9%	0.27
***Included 4 and 5 in response^a^***	***51.9%***	***48.4%***	***0.04***	***52.3%***	***51.2%***	***0.70***
***Answered 4 and 5 only^b^***	***29.1%***	***27.8%***	***0.40***	***29.8%***	***27.9%***	***0.43***

*COVID-19, coronavirus disease 2019; H & L, Health & Life; SD, standard deviation; PH, Public Health*.

a *Proportion of responders who included Statements 4 and 5 in their response along with other statements from the list of options*.

b *Proportion of responders who included none other than Statements 4 and 5 in their response, indicating a positive attitude toward a person with COVID-19*.

c *Sub-group of participants registered in “Health or Life Sciences” programs according to their response with regard to whether epidemic management and Public Health response was covered in the curriculum*.

### Predictors of Knowledge and Positive Attitude Toward Persons With COVID-19

The self-assessment of students regarding their own knowledge was a predictor of objectively assessed knowledge, after adjusting for sociodemographic factors in multivariable stepwise regression models (Table 4). However, the observed difference across tertiles of perceived knowledge was lower than one additional correct question (b coefficient 0.23 per tertile increase, 95% CI 0.17, 0.29; *p* < 0.001). Positive attitude toward persons with COVID-19 was also a predictor of higher knowledge score on average (b coefficient 0.26, 95% CI 0.16–0.36) and of the likelihood to answer eight or more questions correctly (OR 1.30, 95% CI 1.10–1.53). The reverse is also true; the odds of expressing a positive attitude increased by 18% (95% CI 13%, 24%) per unit increase in terms of the knowledge score.

**Table 4:** Predictors of knowledge and positive attitude toward persons with COVID-19 as estimated in stepwise linear and logistic multivariable regression models (*N* = 3,641).

	**Knowledge** **score (0–10)**	**Knowledge** **score ≥8**	**Positive attitude** **(at least)^a^**	**Positive attitude** **(only)^b^**
	**B coefficient** **(95% CI)** ***p*-value**	**Odds ratio** **(95% CI)** ***p*-value**	**Odds ratio** **(95% CI)** ***p*-value**	**Odds ratio** **(95% CI)** ***p*-value**
**Across tertiles of perceived knowledge**	0.23 (0.17, 0.29) *p* < 0.001	1.19 (1.08, 1.32) *p =* 0.001		
**Positive attitude**	0.26 (0.16, 0.36) *p* < 0.001	1.30 (1.10, 1.53) *p =* 0.002		
**Per 1 unit increase in knowledge score**			1.12 (1.08, 1.17) *p* < 0.001	1.18 (1.13, 1.24) *p* < 0.001
**Demographics**				
Language: not Greek	−0.16 (−0.30, −0.01) *p =* 0.03		1.27 (1.05, 1.53) *p =* 0.01	1.47 (1.20, 1.80) *p* < 0.001
Male gender	0.14 (0.03, 0.24) *p =* 0.01	1.28 (1.08, 1.51) *p =* 0.005		0.84 (0.72, 0.98) *p =* 0.03
Study level: Master's	0.35 (0.19, 0.51) *p* < 0.001	1.47 (1.15, 1.89) *p =* 0.002		
Study level: Doctoral	0.77 (0.56, 0.98) *p* < 0.001	1.81 (1.34, 2.46) *p* < 0.001		1.35 (1.01, 1.80) *p =* 0.04
Study level: Other		1.75 (1.05, 2.93) *p =* 0.03		1.73 (1.07, 2.82) *p =* 0.03
Not Health/Life sciences	−0.28 (−0.39, −0.18) *p* < 0.001	0.73 (0.62, 0.86) *p* < 0.001		
**Perceptions and Beliefs**				
Travel restrictions: necessary	0.33 (0.14, 0.53) *p =* 0.001	1.79 (1.23, 2.61) *p =* 0.002		
Fear of spread: same	0.19 (0.09, 0.30) *p* < 0.001	1.38 (1.17, 1.63) *p =* 0.009		1.19 (1.02, 1.38) *p =* 0.03
Antibiotics: know someone	−0.94 (−1.26, −0.63) *p* < 0.001	0.41 (0.21, 0.80) *p* < 0.001		

*CI, confidence interval; COVID-19, coronavirus disease 2019*.

a *Included Statements 4 (“Doesn't change the way I think about them”) and 5 (“I will try to support them as much as I can”) in their response along with other statements*.

b *Included none other than Statements 4 and 5 in their response*.

The program of study was a predictor of knowledge, with a slightly lower score (−0.28 95% CI 0.39, −0.18) among non-Health/Life Science students who were 27% less likely to respond correctly to eight or more questions (OR 0.73 95% CI 0.62, 0.86). In contrast, Health or Life Sciences students were not more likely to express a positive attitude compared to others.

Men and postgraduate students appeared more likely to respond correctly. However, being a man was associated with lower odds of a positive attitude toward persons with COVID-19 (OR 0.84, 95% CI 0.72–0.98). Other than being more knowledgeable, the postgraduate students were also more likely to express a positive attitude. Being a non-native Greek speaker was associated with a marginally lower knowledge score (−0.16 95% CI −0.30, −0.01) but a higher positive attitude (OR 1.47 95% CI 1.20–1.80). Knowing someone who takes antibiotics preventatively for COVID-19 and not considering travel restrictions necessary were negatively associated with knowledge. In fact, these were among the characteristics with the strongest associations. In terms of “fear,” those who believed the spread in Cyprus would be similar to elsewhere were both more knowledgeable and more likely to express a positive attitude toward an infected person (OR 1.19, 95% CI 1.02–1.38).

## Discussion

The current survey collected information on an epidemiologically important population subgroup to evaluate knowledge, perceptions, and attitudes in relation to the COVID-19 pandemic. Students are a socially active population with important implications. The recent resurgence of COVID-19 has been often attributed to the leisure activities of young persons (13). Therefore, findings can be used to tailor and improve the content and quality of the information provided to students, in order to safely maintain their social habits without increasing the risk of SARS-CoV-2 transmission.

Participants assessed their knowledge relatively high, even though gaps (e.g., symptoms and transmission) and misconceptions (e.g., need for protective measures mostly by populations at risk) were identified. While those who assessed their knowledge higher were more likely to respond correctly, the observed differences were small. However, better knowledge was associated with a positive attitude toward persons infected with SARS-CoV-2, while studying at the postgraduate level was a predictor of both knowledge and positive attitude. Interestingly, those who neither underestimated nor overestimated the risk of spread appeared both more knowledgeable and more likely to have a positive attitude.

While Health or Life Science students performed marginally better in terms of knowledge, they were not more likely to express a positive attitude. Only one in the three participants expressed a positive attitude without also projecting any kind of judgment about an infected person. COVID-19-related stigma and reactive behaviors have been documented across the world, with social and health consequences on the recipients of such attitudes (14).

Our study was addressed to all University students across major universities in Cyprus, irrespective of the academic discipline, with one in five students responding to the call. Previous studies among students assessing knowledge and perceptions with regards to COVID-19 often restricted their investigation to a single faculty or University (15–18) or extend their investigation across several universities, with a few exceptions (19–21), often with a focus on students in clinical programs (11, 12, 21–24). Achieved sample sizes vary widely across these studies from as few as 250 to as many as 1,400. While in most cases, sample size exceeds the minimum required based on precision analysis for the estimation of percentages with a 5% margin of error, the representativeness of the sample due to volunteer bias is a common limitation across studies. Studies also differ in focus (i.e., knowledge, attitudes, perceptions, and/or practices) as well as questionnaire content, range, and type of response items (e.g., True/False vs. multiple choice questions), thus, not allowing direct comparisons. Each study should be understood in the local context and timing both in terms of the course of the epidemic as well as the response of the authorities and the role of the media.

Nevertheless, studies denote some common findings. For instance, higher knowledge was associated with a more positive approach toward risks and perceptions related to COVID-19, indicative of higher levels of understanding of risk factors and virulence (19, 20, 23, 24). A median of 6 (out of 10) correct answers was recorded in our survey, which is comparable to or lower than other cohorts (19, 20, 23). Important gaps have been noted in the knowledge of basic modes of transmission. A notable proportion of respondents in several surveys did not recognize respiratory droplets as the main transmission route of the virus (ranging between 8 and 75%, compared to 11% in our study) or did not consider masks (ranging between 10 and 32%, compared to 28% in our study) as an important protective measure (11, 17, 19). It should be noted that at the time of this survey, the use of masks by the general population was not compulsory in Cyprus. Varied awareness of the main symptoms of the disease has also been reported in previous studies. An example here is the triad of fever, cough, and weakness/fatigue, vs. the triad of fever, cough, and shortness of breath, recognized as the most common symptoms of COVID-19 (11, 19, 25) by 8.6 and 63%, respectively.

Certain misconceptions were also detected among our cohort regarding preventive measures of SARS-CoV-2 transmission. In our study, participants were asked if they knew someone who took antibiotics preventatively for COVID-19. Even though <3% of the participants reported positively to this question, this was one of the characteristics with the strongest association with a lower knowledge score. Previous studies which included an antibiotics-related question have also identified that this may have been a common misconception in the early days (16, 18). More importantly, as many as 42% in our study believed that measures should be mostly used by the elderly and populations at risk. This indicates the need to test not only communication messages for consistency but also effect, as the focus to protect the elderly, and the vulnerable groups may inadvertently lead the younger people to underestimate the importance of taking protective measures themselves.

Even though attitudes toward people infected with COVID-19 was not the intended focus that we prioritized of this study, it was notable, based on the data collected, that only one in three respondents adopted both statements tapping on empathy without projecting judgment (i.e., bad self-hygiene) and assigning blame (e.g., negligent). This should also be interpreted in the local context. While reporting in Cyprus may have been largely neutral, focusing on daily updates and the need to adhere to measures, it was not characterized by empathy toward the people affected, and it might have at least indirectly triggered “blame assignment.” Similar studies among University populations do not always include stigma-related questions, thus not allowing direct comparisons. A notable exception is a study by Baniyas et al. (22), where as many as 67% among a sample of 712 medical and Health Science students across universities in the United Arab Emirates reported that infection with the virus is associated with stigma. Similarly, a study of 1,404 students across six medical schools in Jordan found that one in three would prefer to keep it a secret if a family member got infected (11).

In our study, women and postgraduate students were more likely to express a positive attitude, while attitudes of students in Health and Life Science did not differ from those in other programs as also shown by Alzoubi et al. (17). This may not be surprising considering that students in these disciplines do not necessarily have innate levels of empathy (26) or become more empathic during their studies (27), raising questions about the need to strengthen empathy-focused training for future health professionals (28).

To some extent, findings regarding the knowledge of disease symptoms may be attributed to changes in the information provided to the public as the pandemic progressed. For example, 44% of students included pregnant women in the vulnerable groups. Even though pregnant women were not listed in the vulnerable groups by the Ministry of Health, working pregnant women were included in the eligibility list for temporarily suspending their employment and received state funding during the pandemic by the Ministry of Labor. Lastly, some questions may have been difficult for participants to answer correctly (i.e., global fatality rate), due to inconsistent terms used by media.

Certain limitations in this study should be acknowledged. Due to the rapid progress of the pandemic and the need to timely address the issues investigated in this work, a pilot study to pretest the questionnaire for readability and comprehension with the intended audience or a study on student subpopulations was not performed. Nevertheless, the intention was not to develop and validate a composite measurement scale of knowledge for future use but to identify certain misconceptions among the student population regarding distinct but important issues. In fact, opting for multiple-choice questions compared to the common choice of True vs. False, allowed us to explore the range and combinations of responses.

To the best of our knowledge, this is the largest survey to date that assesses knowledge, perceptions, and attitudes of students in relation to COVID-19. Due to the large sample size with national coverage, the study had more than adequate statistical power to detect even small differences among sub-groups. More importantly, including students from all programs, not just clinical programs of study, allowed to assess the extent to which knowledge and attitudes of students in the Health and Life Sciences differ compared to “all others.” Many similar studies with a University student sample either do not explore this information or restrict the survey to medical and other Health Sciences students. The fact that we selected to include only students studying in conventional programs adds further strength to the study, as we sought information only from students whose responses were pertinent to Cyprus.

While the sample represents one in five of all registered students across participating universities, selection (volunteer) bias cannot be excluded. While this may affect the representativeness of the sample, the direction in which it might have affected the results is not clear as students who responded to our call might have been more or less well-informed compared to the overall student community. While the online nature of the survey might have affected the findings in terms of knowledge (e.g., participants could confirm their answers online), it should be noted that relatively large variability was observed, with only 20.6% of the participants responding to eight or more questions correctly while an equally substantial percentage of 15.2% answered correctly only for four or less. Furthermore, while participants regarded themselves as moderately knowledgeable (median 75 on a scale of 0–100, IQR 66–85), the association between self-assessed and actual knowledge was not strong.

The cross-sectional nature of the study does not lend itself to assessing the extent to which knowledge, perceptions, attitudes, and behaviors among the University student population, have changed over time. The study was performed at a point in time when the number of COVID-19 cases in the country was still low. It would be interesting to assess how attitudes of University students toward people infected and affected by COVID-19 have changed since then. A number of studies from Cyprus were identified, albeit with a cross-sectional design. In fact, they tend to restrict their investigation to the knowledge and perceptions of healthcare providers (29) and more recently, attitudes toward COVID-19 vaccines (30, 31). We are not aware of any prospective studies that have tracked knowledge, perceptions, practices, and/or attitudes over time either among the general Greek-Cypriot population or any sub-population groups.

Regarding the practical implications of the study, each University involved in this study actively engaged with its student body with the aim to disseminate up-to-date information using various media and channels, in the form of websites, informational leaflets, fact sheets, FAQ, etc. Furthermore, universities strengthened the direct support offered to the student community at a personal level. This took several forms, such as science cafés, telephone hotlines, or other counseling services. One notable example was the “we are in this together” direct line of the Cyprus University of Technology (32). Even though this was originally conceived as a support service offered to healthcare professionals by the Department of Nursing of the University, records indicated that the majority of calls originated from students and community members.

## Conclusions

The current survey among a large sample of University students denoted specific targets to improve information activities and guidance toward this important population group. Findings highlight the importance of continuous communication, especially as guidance changes, to minimize misunderstandings that may undermine optimal prevention measures in the community. Although future actions should aim to improve knowledge, the various communication channels should be aware of the effect that communication of knowledge may have on shaping attitudes toward infected and affected persons. Factual information and recommendations by international and national authorities have changed during the course of the pandemic, highlighting not only the importance of communication from official channels to cover “what is currently known” but also “what is not known” and how information may still change based on emerging evidence. Other than transparency in the communication, messages, whether originating from regulatory authorities or University channels should also be assessed for their effect. Beyond factual information, communication should tap on empathy and avoid stigma. In Cyprus, while there was some organized community action from institutions and bodies (e.g., Municipalities and Universities), the extent of prosocial behavior at the individual level is not known and should be explored in future studies. Stories of organized community action did not hit the mainstream media to the same extent as elsewhere, while the official media in Cyprus may have inadvertently, at least at the time of this study, triggered “blame assignment.” While entering the first nationwide lockdown and University education moving online, the motivation behind adhering to measures may make little difference if the end goal is to stop the spread of the disease. However, understanding the drivers underpinning attitudes, practices and behaviors is vital when universities are open and social activity resumes. Future studies should focus on other important constructs that mediate the response of the public, such as empathy and compassion toward people affected by COVID-19.

## Data Availability Statement

The raw data supporting the conclusions of this article will be made available by the authors, without undue reservation.

## Author Contributions

ND, GN, and EC conceptualized the study and formed the original research questions. All authors contributed to the finalization of the research questions, the methodological design of the study and the development of the survey questionnaire. NM performed the data analysis and prepared the tables and the first draft of the description of the results. All authors contributed to the interpretation of the data and the writing of the first draft of the manuscript. All authors contributed to the review and editing toward the final draft. All authors read and agreed to the published version of the manuscript.

## Conflict of Interest

The authors declare that the research was conducted in the absence of any commercial or financial relationships that could be construed as a potential conflict of interest.

## Publisher's Note

All claims expressed in this article are solely those of the authors and do not necessarily represent those of their affiliated organizations, or those of the publisher, the editors and the reviewers. Any product that may be evaluated in this article, or claim that may be made by its manufacturer, is not guaranteed or endorsed by the publisher.
