# Supplementary material for: Gaps in Knowledge About SARS-CoV-2 & COVID-19 Among University Students Are Associated With Negative Attitudes Toward People With COVID-19: A Cross-Sectional Study in Cyprus
Source: Front Public Health. 2021 Nov 19;9:758030. doi: 10.3389/fpubh.2021.758030 (PMC8640461; doi:10.3389/fpubh.2021.758030)
Supplement: Supplementary file 1 [file Data_Sheet_1.PDF]

Ερωτηματολόγιο Φοιτητών/τριών της Κύπρου για τον νέο κορωνοϊό COVID-19 (University Student Survey in Cyprus for the new coronavirus COVID-19)

ΠΛΗΡΟΦΟΡΙΕΣ ΓΙΑ ΤΟ ΕΡΩΤΗΜΑΤΟΛΟΓΙΟ

Το ερωτηματολόγιο αυτό αποτελεί κοινή έρευνα ομάδας ακαδημαϊκών από το Πανεπιστήμιο Κύπρου, το Τεχνολογικό Πανεπιστήμιο Κύπρου, το Πανεπιστήμιο Λευκωσίας, το Ευρωπαϊκό Πανεπιστήμιο και το Πανεπιστημίο Frederick.

Στόχος του ερωτηματολογίου είναι να συλλέξει δεδομένα που έχουν σχέση με την ποιότητα της ενημέρωσης/γνώσης των φοιτητών/τριών των Πανεπιστημιακών Ιδρυμάτων που συμμετέχουν για τον νέο κορωνοϊό (COVID-19). Τα δεδομένα του ερωτηματολογίου θα μας βοηθήσουν να αναγνωρίσουμε στοιχεία γνώσης και παρανόησης για τον ιό από την φοιτητική κοινότητα ώστε να σχεδιάσουμε κατάλληλο ενημερωτικό υλικό το οποίο θα βοηθήσει στην αποτροπή εξάπλωσης του ιού.

Το ερωτηματολόγιο περιέχει συνολικά μόνο 19 ερωτήσεις γνώσης και αντίληψης, για τον νέο κορωνοϊό, χωρισμένες σε 3 μέρη. Κάποιες από τις ερωτήσεις θα εμφανισθούν αφού πρώτα απαντήσετε σε συγκεκριμένες ερωτήσεις, για σκοπούς ανάλυσης.Ο συνολικός χρόνος συμπλήρωσης του ερωτηματολογίου αναμένεται να είναι 7-10 λεπτά.

Η συμπλήρωση του ερωτηματολογίου είναι εθελοντική. Αν οποιαδήποτε στιγμή κατά τη διάρκεια του ερωτηματολογίου θελήσετε να ακυρώσετε τη διαδικασία, μπορείτε απλά να κλείσετε το παράθυρο. Κανένα από τα στοιχεία σας δεν αποθηκεύονται στο σύστημα εαν δεν τελειώσετε και υποβάλλετε οριστικά το ερωτηματολόγιο.

Το ερωτηματολόγιο έχει πάρει την έγκριση της Εθνικής Επιτροπής Βιοηθικής Κύπρου, με αριθμό ΕΕΒΚ ΠΚ 2020.01.51.

Οποιαδήποτε διευκρίνηση χρειάζεστε για το ερωτηματολόγιο, τον στόχο του και τις ερωτήσεις που περιλαμβάνει, μπορείτε να επικοινωνήσετε με τον υπεύθυνο της έρευνας, Δρ Νικόλα Διέτη, Επ.Καθ. Φαρμακολογίας Ιατρικής Σχολής Πανεπιστημίου Κύπρου, dietis.nikolas@ucy.ac.cy, τηλ 22895211.

Ευχαριστούμε για την συμμετοχή σας! Η Ερευνητική Ομάδα "COVID19 INTERACT"

INFORMATION FOR THIS QUESTIONNAIRE

This questionnaire is part of a joint research project by academics of the University of Cyprus, the Cyprus University of Technology, the University of Nicosia, the European University and the Frederick University.

The aim of this questionnaire is to collect data related to the quality of knowledge/information that University students in Cyprus have regarding the new coronavirus (COVID-19). The data will help us understand the elements of knowledge and misconception of students regarding the new virus and help us design appropriate information material that may help against the spread of the virus in Cyprus.

The questionnaire contains only 19 questions of knowledge & perceptions for the new coronavirus, in 3 different parts. Some of the questions will pop out only after the answering of specific questions, for analysis purposes. The total time required for the completion of this questionnaire is only 7-10 minutes.

Answering of the questionnaire is voluntary. If you wish to quit the questionnaire at any time, you can do so just by closing the browser window. Your answers and data are not stored in the system unless you complete and submit the questionnaire.

The questionnaire has been approved by the National Bioethics Committee of Cyprus with the number ΕΕΒΚ ΠΚ 2020.01.51.

If you have any questions regarding this questionnaire, its aims and its content, you may contact the Lead Researcher Dr. Nikolas Dietis, Assistant Professor of Pharmacology, University of Cyprus Medical School, dietis.nikolas@ucy.ac.cy, tel 22895211.

Thank you very much for your participation!

The Research Team "COVID19 INTERACT"

Επιβεβαίωση Συμμετέχοντα
Participant Confirmation

Ποιο είναι το email που έχεις στο Πανεπιστήμιό σου;

What is your University email?

____

Είναι σημαντικό οι απαντήσεις σου στις παρακάτω ερωτήσεις να είναι ειλικρινείς και να μην χρησιμοποιείς εξωτερικό ενημερωτικό υλικό για να απαντήσεις. Στόχος μας είναι η καταγραφή της πραγματικής γνώσης & αντίληψης των φοιτητών/τριών μας για να σχεδιάσουμε πιο αποτελεσματική ενημέρωση.

It is important that your answers to the following questions are sincere and that you don't use external information sources to answer the questions here. Our aim is to record the actual knowledge & perception of our students in order to design more effective measures of information.

◯ ΝΑΙ, ΚΑΤΑΛΑΒΑΙΝΩ (YES, I UNDERSTAND)

ΜΕΡΟΣ Α: Χαρακτηριστικά Συμμετέχοντα (8 ερωτήσεις)

PART A: Participant Characteristics (8 questions)

1 Ποιο ήταν το φύλο σου κατά τη γέννηση;

What gender were you assigned at birth?

- ◯ Θήλυ (Female)
- ◯ Άρρεν (Male)
- ◯ Άλλο (Other)
- ◯ Δεν θέλω να απαντήσω (I don't want to answer)

1 Ποιο είναι το επίπεδο του προγράμματος σπουδών που είσαι εγγεγραμμένος/η στο Πανεπιστήμιό σου;

What is the level of the program of study you are currently enrolled in your University?

- ◯ Προπτυχιακό (Bachelor)
- ◯ Μεταπτυχιακό (Master)
- ◯ Διδακτορικό (Doctorate)
- ◯ Άλλο (Other)

Σε ποιο Πανεπιστήμιο σπουδάζεις;

In which University do you study?

- ◯ Πανεπιστήμιο Κύπρου (University of Cyprus)
- ◯ Τεχνολογικό Πανεπιστήμιο Κύπρου (Cyprus University of Technology)
- ◯ Πανεπιστήμιο Λευκωσίας (University of Nicosia)
- ◯ Ευρωπαϊκό Πανεπιστήμιο (European University)
- ◯ Πανεπιστήμιο Frederick (Frederick University)

Είναι η μητρική σου γλώσσα τα Ελληνικά;

Is your native language Greek?

- ◯ Ναι (Yes)
- ◯ Όχι (No)

Έχει σχέση το πρόγραμμα σπουδών σου με Επιστήμες Υγείας ή Ζωής; πχ. Ιατρική, Νοσηλευτική, Φαρμακευτική, Βιολογία, Μικροβιολογία, Δημόσια Υγεία κτλ

Is your degree related to Health or Life Sciences? i.e. Medicine, Nursery, Pharmacy, Biology, Microbiology, Public Health etc

- ◯ Ναι (Yes)
- ◯ Όχι (No)

Έχεις διδαχθεί ή θα διδαχθείς, ως μέρος της διδακτικής ύλης του προγράμματος σπουδών μου, για τους τρόπους διαχείρισης επιδημιών/πανδημιών και προστασίας της δημόσιας υγείας;<br><br>Have you been taught or will be taught, as part of the curriculum of your program of study, about the ways of managing epidemics/pandemics and the ways of protecting public health?	◯ Ναι (Yes)<br>◯ Όχι (No)
Έχεις κάποιο συγγενικό σου πρόσωπο, πρώτου βαθμού (σύζυγοι, γονείς, αδέρφια, παιδιά) που είναι ιατρός ή νοσηλευτής/τρια ή φαρμακοποιός ή άλλου είδους επαγγελματίας υγείας; (συμπεριλαμβάνονται και όσοι/ες είναι ακόμα σε στάδιο εκπαίδευσης)<br><br>Do you have first-degree relatives (parents, siblings, spouses, kids) who are medical doctors or nurses or pharmacist or other type of health professionals? (including trainees)	◯ Ναι, έχω (Yes I do)<br>◯ Όχι, δεν έχω (No I do not)<br>◯ Δεν είμαι σίγουρος/η (I am not sure)
Πόσο καλά ενημερωμένος/η πιστεύεις ότι είσαι σχετικά με τον νέο κορωνοϊό (COVID-19); Μετακίνησε τον δείκτη δεξιά ή αριστερά για να δώσεις μια απάντηση σχετική σε ποσοστό ικανοποιητικής πληροφόρησης %.<br><br>How well informed do you believe you are regarding the new coronavirus (COVID-19)? Move the slider left or right to give an answer related to satisfactory information as %.	Καθόλου καλά ρημένος/η (Not well informed) — Πολύ καλά ρημένος/η (Very well informed)<br><br>*(Place a mark on the scale above)*

ΜΕΡΟΣ Β (7 ΕΡΩΤΗΣΕΙΣ)
PART B (7 QUESTIONS)

Γνωρίζεις τα 3 κύρια χαρακτηριστικά συμπτώματα της νόσου COVID-19 (νέος κορωνοιός);<br><br>Do you know the 3 main characteristic symptoms of the disease COVID-19 (new coronavirus)?	◯ Ναι (Yes)<br>◯ Όχι (No)<br>◯ Δεν είμαι σίγουρος/η (I'm not sure)
Ποια είναι τα τρία κύρια χαρακτηριστικά συμπτώματα της νόσου COVID-19 (νέος κωρονοϊός);<br><br>What are the three main characteristic symptoms of the disease COVID-19 (new coronavirus)?	☐ Πονοκέφαλος (headache)<br>☐ Πυρετός (fever)<br>☐ Δύσπονοια (shortness of breath)<br>☐ Πόνος στο στήθος (chest pain)<br>☐ Βήχας (cough)<br>☐ Διάρροια (diarrhea)<br>☐ Αδυναμία (weakness/fatigue)<br>☐ Καταρροή (running nose)
Ξέρεις πώς μεταδίδεται ο νέος κορωνοϊός (COVID-19);<br><br>Do you know how is the new coronavirus (COVID-19) transmitted?	◯ Ναι (Yes)<br>◯ Όχι (No)<br>◯ Δεν είμαι σίγουρος/η (I am not sure)
Με ποιούς τρόπους μπορεί να μεταδοθεί ο νέος κορωνοϊός (COVID-19); Διάλεξε όσα πιστεύεις ότι ισχύουν.<br><br>Through which ways can the new coronavirus (COVID-19) be transmitted? Choose any you believe is true.	☐ Μέσω σεξουαλικής επαφής (sexual contact)<br>☐ Μέσω σταγονιδίων που βγαίνουν από φτάρνισμα/βήξιμο και επαφή με στόμα/μάτια/μύτη (through droplets from sneezing/coughing and contact with mouth/eyes/nose)<br>☐ Μέσω εισπνόμενου αέρα (through breathing air)<br>☐ Μέσω κατανάλωσης/κατάποσης μολυσμένων τροφών (consuming/eating contaminated food)<br>☐ Μέσω επαφής με μολυσμένη επιφάνεια και κατόπιν άγγιγμα στόμα/μάτια/μύτη (through contact with a contaminated surface and then touching mouth/eyes/nose)

Πόσος είναι ο μέγιστος χρόνος που μπορεί να μεσολαβεί μεταξύ της μόλυνσης από τον νέο κορωνοϊό (COVID-19) και την εμφάνιση των πρώτων συμπτωμάτων;<br><br>What is the maximum time that can be between the time of infection with the new coronavirus (COVID-19) and the first presentation of symptoms?	◯ Μέσα σε 1 μήνα (Within 1 month)<br>◯ Μέσα σε 2 εβδομάδες (Within 2 weeks)<br>◯ Μέσα σε 1 εβδομάδα (Within 1 week)<br>◯ Μέσα σε 2 ημέρες (Within 2 days)
Ποιο πιστεύετε ότι είναι το μέσο παγκόσμιο ποσοστό θνητότητας του νέου κορωνοϊού (COVID-19), δλδ ο αριθμός θανάτων ανά 100 μολυσμένους με τον ιό.<br><br>What do you think is the average global percentage of fatality of the new coronavirus (COVID-19), i.e. the number of deaths per 100 infected with the virus.	◯ Μεταξύ 1-10% (Between 1-10%)<br>◯ Μεταξύ 10-30% (Between 10-30%)<br>◯ Πάνω από 30% (Over 30%)<br>◯ Δεν γνωρίζω (I don't know)
Ποιες ομάδες ανθρώπων πιστεύετε ότι είναι οι ευπαθείς ομάδες, δλδ αυτές που έχουν μεγάλο ρίσκο να αρρωστήσουν βαριά ή να πεθάνουν από την μόλυνση με τον νέο κορωνοϊό (COVID-19);<br><br>Which groups of people you think are the vulnerable groups, i.e. those that have a high risk of getting seriously sick or die from infection with the new coronavirus (COVID-19)?	☐ Παιδιά (Children)<br>☐ Άνθρωποι άνω 65 ετών (People over 65 year old)<br>☐ Έγκυες γυναίκες (Pregnant women)<br>☐ Άνθρωποι με χρόνιες ασθένειες (People with a chronic disease)<br>☐ Μετανάστες/πρόσφυγες (Migrants/Refugees)<br>☐ Άνθρωποι με χαμηλό ανοσοποιητικό σύστημα (People with low immune system)

ΜΕΡΟΣ Γ (4 ΕΡΩΤΗΣΕΙΣ)
PART C (4 QUESTIONS)

Επέλεξε αν πιστεύεις ότι αληθεύει ή όχι, κάθε μία από τις παρακάτω δηλώσεις για τον νέο κορωνοϊό (COVID-19).
Select for the statements below for the new coronavirus (COVID-19), whether they are True or False.

	Αληθεύει (True)	Δεν αληθεύει (False)	Δεν είμαι σίγουρος/η (I am not sure)
Μειώνουμε την πιθανότητα μόλυνσης από τον νέο κορωνοϊό εαν πλύνουμε καλά τα χέρια μας με νερό και σαπούνι (We reduce the risk of infection by the new coronavirus if we wash our hands thoroughly with water and soap)	○	○	○
Μέτρα προστασίας από τον νέο κορωνοϊό οφείλουν να τηρούν κυρίως οι ηλικιωμένοι και οι ευπαθείς ομάδες (Protective measures for the new coronavirus COVID-19 should be mostly used by the elderly and the populations at risk)	○	○	○
Η χρήση απλής χειρουργικής μάσκας από τους υγιείς θεωρείται απαραίτητο μέτρο ατομικής προστασίας από μόλυνση από τον νέο κορωνοϊό (The use of a surgery mask by the healthy is regarded as a necessary protective measure against infection by the new coronavirus COVID-19)	○	○	○

Υπάρχουν κάποια φάρμακα ή εμβόλια που μπορεί να χρησιμοποιηθούν προληπτικά για να μειωθεί η όποια πιθανότητα μόλυνσης από τον νέο κορωνοϊό (There are some medicines or vaccines that can be taken preventively to reduce any potential risk of being infected by the new coronavirus)	○	○	○

ΜΕΡΟΣ Δ (8 ΕΡΩΤΗΣΕΙΣ)
PART D (8 QUESTIONS)

Γνωρίζεις κάποιον/κάποια που παίρνει αντιβιοτικά προληπτικά, για να μειώσει τον κίνδυνο μόλυνσης από τον νέο κορωνοϊό (COVID-19).

Do you know a person who takes antibiotics as a means of prevention of infection from the new coronavirus (COVID-19).

- ◯ Yes
- ◯ No

Πιστεύεις ότι οι αυστηροί ταξιδιωτικοί περιορισμοί (πχ απαγόρευση εισόδου μη-Κυπρίων πολιτών, αναγκαστικός έλεγχος/αυτοπεριορισμός/καραντίνας όσων εισέρχονται) είναι απαραίτητο μέτρο για την μείωση ρυθμού των κρουσμάτων [illegible]

Do you believe that strict traveling restrictions (i.e. prohibition of non-Cypriots from entering, compulsory screening/self-containment/quarantine to anyone entering) is a necessary measure for reducing the rates of new cases of the new coronavirus (COVID-19) infection?

- ◯ Ναι, είναι απαραίτητοι (Yes, they are necessary)
- ◯ Όχι δεν είναι απαραίτητοι (No, they are not necessary)
- ◯ Δεν είμαι σίγουρος/η (I am not sure)

Πιστεύεις ότι η Κύπρος θα έχει περισσότερη, λιγότερη ή ίδια εξάπλωση του νέου κορωνοϊού αναλογικά με τον πληθυσμό της, σε σχέση με τον μέσο όρο της Ευρώπης;

Do you believe Cyprus will have more, less or same spread of the new coronavirus relative to its population, in comparison with the average number in Europe?

- ◯ Περισσότερη (More)
- ◯ Λιγότερη (Less)
- ◯ Ίδια (Same)

Θα επηρέαζε τη σχέση σου ή την άποψή σου για ένα άτομο, η μολυνσή του από τον νέο κορωνοϊό (COVID-19);<br><br>Would a person's infection by the new coronavirus (COVID-19) affect your relationship with or opinion for this person?	◯ Όχι, δεν θα επηρέαζε ούτε τη σχέση μου ούτε την άποψή μου (No, it would neither affect my relationship nor my opinion)<br>◯ Δεν θα επηρέαζε την άποψή μου, αλλά θα επηρέαζε τη σχέση μου μελλοντικά (It would not affect my opinion, but it would affect my relationship in the future)<br>◯ Ναι, θα επηρέαζε την σχέση μου και την άποψή μου (Yes, It would affect my relationship and my opinion)
Μαθαίνεις ότι κάποιος γνωστός σας έχει μολυνθεί από τον νέο κορωνοϊό (COVID-19). Ποιες από τις επιλογές εδώ ισχύουν για εσάς; Επέλεξε όσα νομίζεις ότι ισχύουν για εσένα.<br><br>You find out that a person you know has been infected by the new coronavirus (COVID-19). Which of the choices you support? Choose any that you believe are true for you.	☐ Είχε κακή ατομική υγιεινή (He/she had a bad self-hygiene)<br>☐ Αμέλησε να προστατευτεί σωστά (He/she was negligent to self-protect efficiently)<br>☐ Θα προσπαθήσω να του συμπαρασταθώ όσο μπορώ (I will try to support him/her as much as I can)<br>☐ Στο μέλλον πιθανώς να αποφύγω την συχνή επαφή μαζί του/της (In the future I will probably avoid close conduct with him/her)<br>☐ Δεν αλλάζει κάτι στο τι πιστεύω για αυτόν/αυτήν (It doesn't change the way I think about him/her)
Πιστεύεις ότι το Πανεπιστήμιό σου θα βρει τους τρόπους να ελαχιστοποιήσει την επιρροή που θα έχει η πανδημία στις σπουδές σου;<br><br>Do you believe that your University will find the ways to minimize the impact of the pandemic to your studies?	◯ Ναι (Yes)<br>◯ Όχι (No)<br>◯ Δεν είμαι σίγουρος/η (I am not sure)
Σε ποιο τηλεφωνικό νούμερο μπορείς να επικοινωνήσεις με τις αρχές σχετικά με τον νέο κορωνοϊό (COVID-19);<br><br>What is the telephone number that you can call to contact the authorities regarding the new coronavirus (COVID-19)?	◯ 112<br>◯ 142<br>◯ 1400<br>◯ 1420<br>◯ Δεν θυμάμαι (I don't remember)

Για ποιά θέματα είναι απαραίτητο να επικοινωνείτε με τον αριθμό ανάγκης για τον νέο κορωνοϊό (COVID-19); Διαλέξτε όσα πιστεύετε ότι ισχύουν.

For which reasons is it necessary to contact with the number of emergency for the new coronavirus (COVID-19)? Choose any that you believe are true.

- ☐ Αν έχω συμπτώματα της μόλυνσης (If I have symptoms of the infection)
- ☐ Αν έχω συμπτώματα για περισσότερες από 7 ημέρες (If I have symptoms of the infection for more than 7 days)
- ☐ Αν κάποιος γνωστός μου έχει συμπτώματα της μόλυνσης (if someone I know has symptoms of the infection)
- ☐ Αν έχω συμπτώματα της μόλυνσης και έχω πρόσφατα ταξιδέψει στο εξωτερικό (If I have symptoms of the infection and I recently travelled abroad)
- ☐ Αν θέλω να μάθω για τα συμπτώματα της μόλυνσης (To be informed about the symptoms of the infection)
- ☐ Αν θέλω να μάθω για το πως μεταδίδεται ο ιός (To be informed about the transmission of the virus)
- ☐ Αν χρειάζομαι γιατρό επειγόντως (If I have a medical emergency)

Εθελοντική Συγκατάθεση για μελλοντική επικοινωνία
Voluntary Consent for furure communication

Μας δίνετε την συγκατάθεσή σας να σας στείλουμε μελλοντικά ένα email με πιθανό ερωτηματολόγιο ως συνέχεια αυτής της έρευνας; Η συμμετοχή σας σε πιθανό μελλοντικό ερωτηματολόγιο θα είναι εθελοντική και θα μπορείτε να την απορρίψετε μελλοντικά είτε απαντήσετε θετικά τώρα είτε όχι.<br><br>Do you give us your consent to email you in the future with a potential questionnaire as a continuation of this research? Your participation in a potential future questionnaire will be voluntary and you may reject it later even if you do answer "yes" in this question now or not.	◯ Yes<br>◯ No
